# Ultrahigh volumetric capacitance and cyclic stability of fluorine and nitrogen co-doped carbon microspheres

**DOI:** 10.1038/ncomms9503

**Published:** 2015-09-29

**Authors:** Junshuang Zhou, Jie Lian, Li Hou, Junchuan Zhang, Huiyang Gou, Meirong Xia, Yufeng Zhao, Timothy A. Strobel, Lu Tao, Faming Gao

**Affiliations:** 1 Key Laboratory of Applied Chemistry, College of Environmental and Chemical Engineering, Yanshan University, Qinhuangdao 066004, China; 2 Department of Mechanical, Aerospace, and Nuclear Engineering, Rensselaer Polytechnic Institute, Troy, New York 12180, USA; 3 Geophysical Laboratory, Carnegie Institution of Washington, Washington DC 20015, USA

## Abstract

Highly porous nanostructures with large surface areas are typically employed for electrical double-layer capacitors to improve gravimetric energy storage capacity; however, high surface area carbon-based electrodes result in poor volumetric capacitance because of the low packing density of porous materials. Here, we demonstrate ultrahigh volumetric capacitance of 521 F cm^−3^ in aqueous electrolytes for non-porous carbon microsphere electrodes co-doped with fluorine and nitrogen synthesized by low-temperature solvothermal route, rivaling expensive RuO_2_ or MnO_2_ pseudo-capacitors. The new electrodes also exhibit excellent cyclic stability without capacitance loss after 10,000 cycles in both acidic and basic electrolytes at a high charge current of 5 A g^−1^. This work provides a new approach for designing high-performance electrodes with exceptional volumetric capacitance with high mass loadings and charge rates for long-lived electrochemical energy storage systems.

## Abstract (web summary)

Carbon-based supercapacitors often suffer from poor volumetric capacitance due to the low packing density which arises from attempts to increase the electrode surface area. Here, in contrast, the authors fabricate N and F co-doped non-porous solid carbon spheres and achieve exceptional performances.

## Graphical abstract

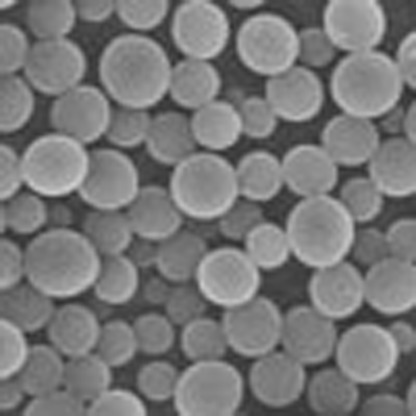

Supercapacitors are promising for applications requiring a large rapid pulse of energy or high power with quick repetitive recharging because of their exceptional power density and cyclability relative to Li batteries^1^,^2^,^3^,^4^,^5^,^6^,^7^,^8^,^9^,^10^,^11^,^12^,^13^,^14^,^15^,^16^,^17^,^18^. The tremendous growth of high-power apparatuses, such as hybrid electric vehicles, has prompted urgent and increased demand for high-performance supercapacitors. Generally, electrical double-layer capacitors, storing energy by adsorbing anions and cations at an electrode/electrolyte interface, have limited capacity and thus energy density. Psuedo-capacitors, such as RuO_2_, MnO_2_ and electrically conducting polymers, store charges based on surface redox reactions, and exhibit specific area-based capacitances 10 times greater than electrical double-layer capacitors. However, pseudo-capacitors suffer from poor stabilities (<10,000 charge/discharge cycles) or high capacitance decay rates.

To enhance the capacitance of electrochemical capacitors (ECs), a typical strategy is to utilize carbon electrodes with large surface areas and appropriate pore-size distributions^19^,^20^. When used as electrodes for symmetric supercapacitor devices, carbon-based materials with high gravimetric surface areas, including activated carbon^21^, carbon nanotubes^22^, carbon spheres^23^,^24^,^25^, templated porous carbons^26^ and graphene^27^,^28^, demonstrate high gravimetric capacitances (up to 250 F g^−1^). However, due to the low packing density (<0.75 g cm^−3^) for porous electrodes, the specific volumetric capacitance, *C*_vol_, a more reliable parameter in evaluating the efficiency of a material for EC energy storage^7^,^29^, is <200 F cm^−3^. To improve capacitive performance, Yang *et al*.^13^ recently reported an irreversible method for chemically converting graphene hydrogel films by capillary pressure to increase the packing density from 0.13 to 1.3 g cm^−3^. The highly compact graphene films (1.33 g cm^−3^) yielded a *C*_vol_ of 255.5 F cm^−3^ in aqueous electrolyte at 0.1 A g^−1^. Its specific capacitance was much higher than those of the existing porous carbon materials, however, is still not sufficient for independent energy storage devices.

Surface functional groups containing oxygen, nitrogen, fluorine or phosphorus can considerably enhance the capacitance of carbon electrodes as a result of the pseudocapacitance effect involved in charge or mass transfer between electrodes and electrolytes^30^,^31^,^32^,^33^. Nitrogen doping has been demonstrated to induce pseudo-capacitance by improving the charge mobility of negative charges on carbon surfaces, thus greatly improving the capacitance. Fluorination is also an effective approach to improve electrochemical capacitive performance of carbon electrodes as a result of increased electrical conductivity because of the semi-ionic bonding features in non-aqueous electrolytes, enhanced polarization from the highly electronegative fluorine functional groups and the refinement of pore structures/surfaces^34^,^35^. However, previously reported volumetric performance for heteroatom-enriched carbon materials is still low because of their relatively low densities and large surface areas^36^.

Here we report a completely different strategy to develop high-performance electrodes with ultrahigh volumetric capacitance and exceptional cyclic stability for electrochemical energy storage. In contrast to conventional wisdom, in which highly porous and large surface area carbon electrodes are utilized, we employ nitrogen and fluorine co-doped carbon microspheres (CM-NF) as electrodes, which were prepared by a simple low-temperature solvothermal route without post-processing. These microspheres are nominally non-porous with Barrett–Emmett–Teller (BET) surface areas as low as 1.4 m^2^ g^−1^, and thus a very-high packing density. In the fluorine and nitrogen co-doped carbon microspheres (CMs), the fluorine doping could have an acidic character, behaving as electron acceptors; meanwhile, nitrogen doping generally provides basic characteristics, inducing electron-donor properties. These active centres may contribute to pseudo-capacitance through additional Faradaic interactions^33^. As such, the synergistic effects of the nitrogen and fluorine co-doping enable the CMs with an ultrahigh specific volumetric capacitance up to 521 F cm^−3^ in H_2_SO_4_, significantly greater than other carbon materials and rival to RuO_2_ or MnO_2_ composites. The CM-NF electrode exhibits an excellent electrochemical stability with no loss of the specific capacitance over 10,000 cycles in a high charge rate of 5 A g^−1^ in both H_2_SO_4_ and KOH. The volumetric capacitance of the co-doped microsphere electrode exceeds 240 F cm^−3^ in basic KOH electrolyte with a very-high mass loading of 16 mg cm^−2^. Unlike highly porous and hollow carbons, these ostensibly nonporous microspheres of carbon have not previously been considered for use as an electrode of supercapacitor due to very-low specific surface area (1.4 m^2^ g^−1^). The superior electrochemical performance and cyclic stability of these CMs are very promising for the development of compact, high-performance supercapacitors.

## Results

### Structural features and composition

**Figure 1:**
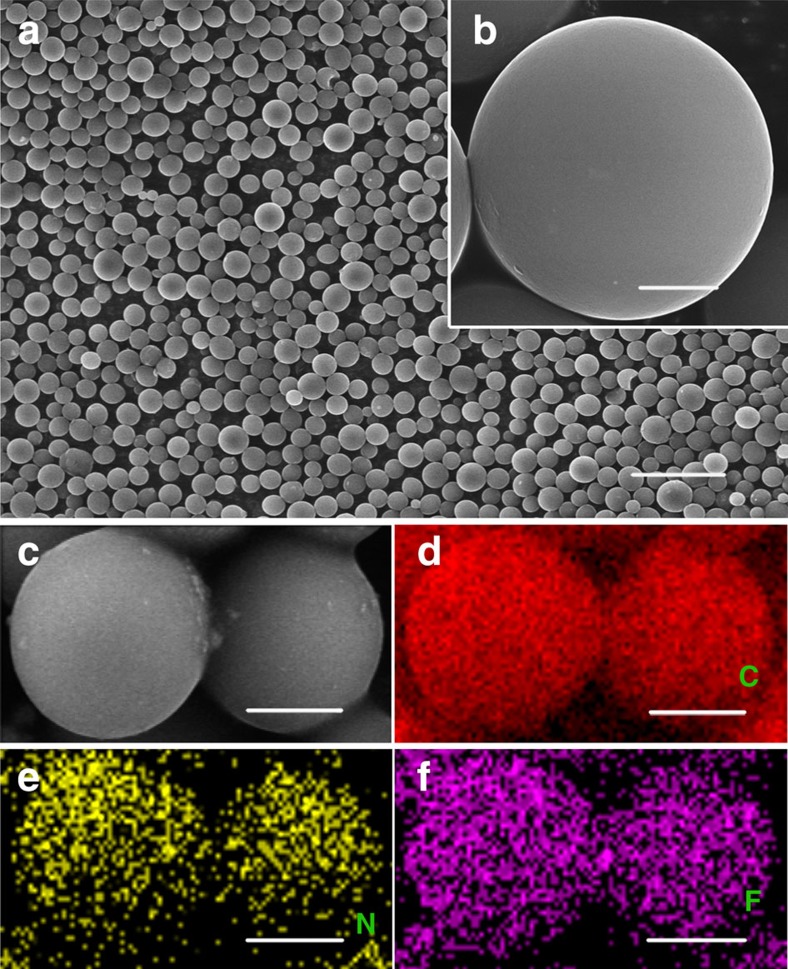
Morphology and elemental distribution of CM-NF electrodes. (**a**) low-magnification s.e.m. image of the CM-NFs; (**b**) high-magnification s.e.m. image showing spherical morphology of CM-NF; (**c**) a s.e.m. image of CM-NFs, and the corresponding EDS elemental mappings of (**d**) carbon (red); (**e**) nitrogen (yellow); and (**f**) fluorine (purple). Scale bar, 15 μm (**a**), 1 μm (**b**) and 2 μm (**c**–**f**).

The CM-NF sphere electrodes and simultaneously incorporation of nitrogen and fluorine were prepared by a simple low-temperature solvothermal route without post-processing. For performance comparison, commercially available solid carbon spheres (purchased from Tianjin BTR New Energy Materials Co, Ltd, China) are used as control materials. Figure 1a shows the morphology of our CM-NF microspheres displaying monodisperse and a uniform diameter of ∼4 μm, and a closeup image (Fig. 1b) shows the smooth surface and nearly-perfect spherical geometry of the CM-NF, and the scanning electron microscopy image (Supplementary Fig. 1a) of a fractured microsphere confirms their overall nonporous nature. Energy-dispersive X-ray spectroscopy and elemental mapping (Fig. 1c–f) of the CM-NF were performed to quantify a uniform distribution of C, F and N species within CM-NF spheres. A high-resolution transmission electron microscopy image and the selected-area diffraction pattern (Supplementary Fig. 2) reveal a highly disordered structure with nanocrystallites as revealed by the (002) graphitic layers.

**Figure 2:**
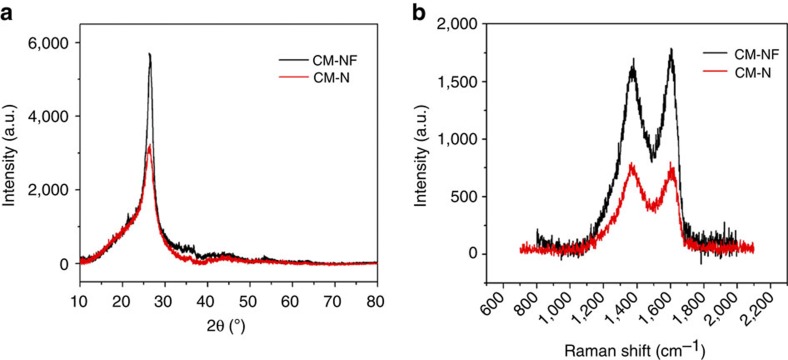
Structural characterization of the carbon microspheres. (**a**) XRD patterns and (**b**) Raman spectra of the CM-NFs and CM-Ns.

Typical X-ray diffraction (XRD) patterns of the CM-NF (co-doping of nitrogen and fluorine) and CM-N (by nitrogen only) (Fig. 2a) show two broadened diffraction peaks at 2-theta of 26.2° and 43.8°, analogous to the (002) and (101) planes of graphite, indicating dominant features of amorphous carbon. For CM-NF sample, the higher (002) peak intensity and reduced peak broadening suggest a greater degree of graphitization for CM-NF than CM-N. Raman spectra (Fig. 2b) show a typical D-band located ∼1,372 cm^−1^ and a G-band ∼1,601 cm^−1^, attributed to the vibration of disordered carbon atoms with defects and *sp*^2^-bonded carbon atoms in a two-dimensional hexagonal lattice, respectively^37^,^38^. The *I*_D_/*I*_G_ (0.88) ratio of CM-NF was lower than that of CM-N (1.01), indicating a larger graphitized ratio in CM-NF, consistent with the XRD results. As compared with the commercial pure CM spheres (see XRD and Raman spectra in Supplementary Fig. 3), significant defects exist in our doped CM-NF spheres, which is beneficial for improving electrochemical performance (see below).

**Figure 3:**
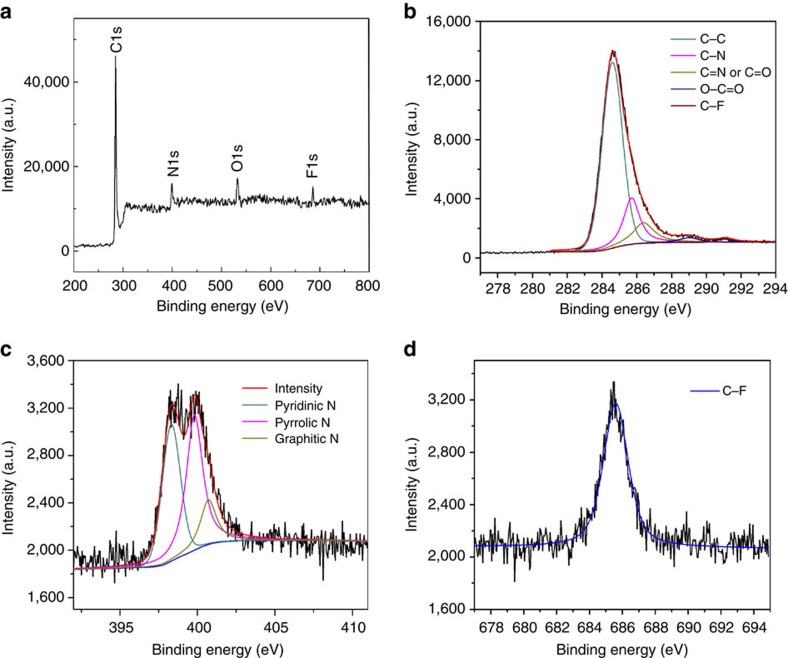
Chemical analysis of the carbon microspheres. (**a**) XPS survey spectrum of CM-NFs; and (**b**–**d**) high-resolution XPS spectra of C 1 s, N 1 s and F 1 s of CM-NFs, respectively.

Elemental chemical states of the CM-NF were further investigated by X-ray photoelectron spectroscopy (XPS) (Fig. 3), showing a predominant narrow graphitic C 1s peak at 284.6 eV. The peaks at 285.7, 286.39, 289 and 291.1 eV are attributed to C–N, C=N, O–C=O, C–F configurations, respectively^39^,^40^. The presence of an O1s peak around 532 eV in CM-NF is presumably due to partial surface oxidation^41^. The complex N1s spectra could be further deconvoluted into three different peaks at the binding energies of 398.3, 399.8 and 400.7 eV, attributable to the pyridinic N, pyrrolic N, graphitic N, respectively. Pyridinic N (N1) atoms, located at the edges of graphitic planes, bond to two carbon atoms and hybridize one *p*-electron to the aromatic system. Pyrrolic-N (N2) atoms bond with two carbon atoms and contribute to the *p* system with two *p*-electrons. Graphitic N atoms are incorporated into the graphene layer and substitute carbon atoms within the graphene plane. The corresponding F1s XPS spectrum (Fig. 3d) shows a single peak centred ∼685.6 eV corresponding to semi-ionic C–F^40^. The XPS spectrum of the CM-N (see Supplementary Fig. 4) shows a similar pattern with a distinct peak at 284.6 eV and small peaks at 398.8 and 532.3 eV, corresponding to C, N, O configurations, respectively. Quantitative elemental analysis indicates a high doping content of nitrogen in CM-N (9.89 wt%), and the N and F elemental analysis of the CM-NF yields 8.76 wt% and 3.4 wt%, respectively (See Supplementary Table 1). The CM-NF and the corresponding CM-N and commercial pure CM carbon materials have very-low surface areas of 1.4, 1.9 and 1.0 m^2^ g^−1^, respectively. The CM-NF displays a typical type II isotherm based on the adsorption/desorption isotherms (Supplementary Fig. 5a), characteristic of macroporous or non-porous materials.

To overcome the limitations of nitrogen sorption method in measuring the pores with the size <1 nm, CO_2_ adsorption at 273.2 K was performed to assess the ultramicropores. The presence of ultramicropores in the ∼0.55 nm size range can be seen from the CO_2_ sorption data (Supplementary Fig. 5a,b), and a total pore volume of ∼0.015 cm^3^ g^−1^ is confirmed. Porosity measurement with data from small-angle X-ray scattering (Supplementary Fig. 5c) also shows evidence of nonporous nature in the synthesized CM-NF. The existence of ultramicropores within the carbon spheres may be beneficial for enhancing the ionic transport behaviour, and thus improve the electrochemical performance as supercapacitor electrodes. Smaller size pores in the Angstrom range may exist in the CM-NF spheres that may not be accessible to gases, even CO_2_, but could allow penetration of small ions, for example, K^+^ and H^+^, further improving the electrochemical performance of electrodes.

### Electrochemical performance

**Figure 4:**
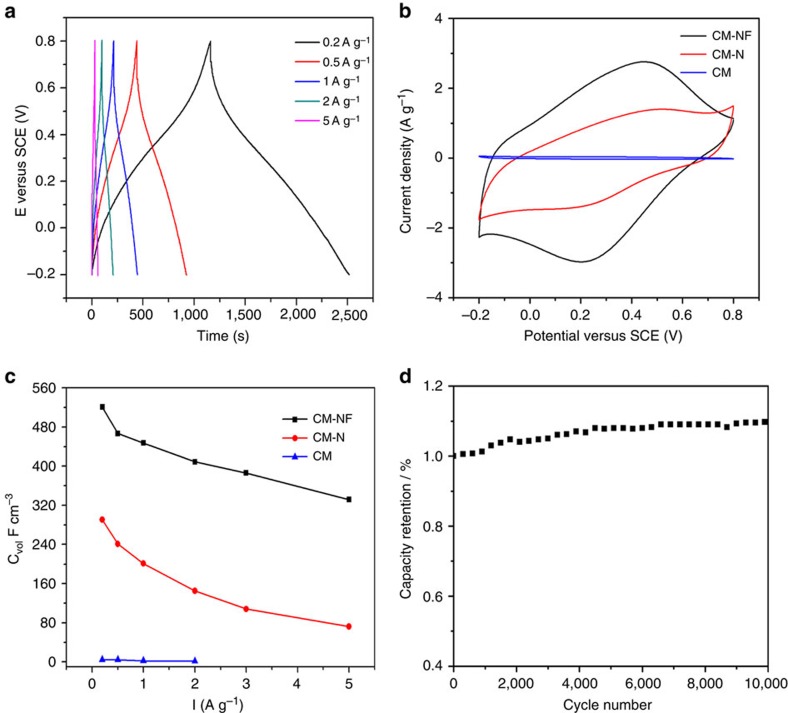
Electrochemical performance of the carbon microspheres. (**a**) galvanostatic charge/discharge curves of CM-NFs samples in 1 M H_2_SO_4_ solution with different current densities; (**b**) CV curves of CMs, CM-Ns and CM-NFs samples in 1 M H_2_SO_4_ solution at a scan rate of 10 mV s^−1^; (**c**) corresponding capacity retentions at the current density from 0.1 to 5 A g^−1^; and (**d**) stability evaluation of the CM-NF electrodes in 1 M H_2_SO_4_ solution at a charge current of 5 A g^−1^ for the 10,000 cycles.

The electrochememical performance of the co-doped carbon spheres was evaluated by galvanostatic charge-discharge (Fig. 4a and Supplementary Fig. 6b) and cyclic voltammograms (CV) (Fig. 4b and Supplementary Fig. 6a) at various current densities, tested in a three-electrode cell in 1 mol l^−1^ H_2_SO_4_ or 6 mol l^−1^ KOH at a scan rate of 10 mV s^−1^. For comparison, the electrochemical performance of the control electrode based on commercial carbon spheres was also tested (Fig. 4b), and a small rectangular curve and very-low capacitance are observed for the commercial CMs. For CM-NF, a distorted rectangular-like shape with distinct pseudocapacitive behaviour over a wide voltage range of −0.1–0.6 V (versus saturated calomel electrode (SCE)) was identified (Fig. 4b), attributed to Faradaic redox reactions of the nitrogen and fluorine dopants^42^. Particularly, a distinct pair of broad and overlapping redox peaks were clearly observed in the CV curve and are more obvious in acidic than in basic electrolytes, probably because of the basic character of the dopants in these carbon materials^33^. Compared with CM-N, CM-NF exhibited capacitive behaviour with the appearance of a significantly larger rectangular-like shape in CV curves, indicating a greatly increased capacitance due to the F doping. Doping N and F in the CM could not only enhance the surface wettability between electrolyte and electrode materials, but could also participate in the pseudo-capacitance reaction^43^.

The redox reactions could also be observed in the galvanostatic charge/discharge curves (Fig. 4a and Supplementary Fig. 6b), in which a nonlinearity occurred revealing representative pseudo-capacitive behaviours of the samples. A small transition in line slopes around −0.3 V in the basic solution is correlated with the redox peaks in the CV curves. The co-doping of nitrogen and fluorine greatly improves the specific capacitance of the CM electrodes, as calculated based on the charge/discharge curves. The CM-NF electrode possesses a specific capacitance approaching 270 F g^−1^ at a current density of 0.2 A g^−1^ in the acid solution, representing an 80% increase over CM-N (153 F g^−1^), and two orders of magnitude greater than commercial CM without heteroatom doping (2 F g^−1^). The CM-NF electrode displays an ultrahigh volumetric capacity up to 521 F cm^−3^ at 0.2 A g^−1^ (Fig. 4c), the highest capacity ever reported for carbon-based electrodes. The volumetric capacitance of the co-doped electrode is 100% greater than that of the CM-N with nitrogen doping only (262 F cm^−3^). Three-electrode and two-electrode supercapacitor cells (Supplementary Fig. 6) were constructed to assess the electrochemical performance in 6 M KOH solution. The redox reactions could also be observed in the galvanostatic charge/discharge curves. The single CM-NF electrode in basic solution also demonstrates a high volumetric capacitance of 365 F cm^−3^ (a three-electrode cell) and 328 F cm^−3^ (a two-electrode cell) at a current density of 0.1 A g^−1^, as calculated based on the charge/discharge curves, confirming the excellent electrochemical properties of the synthesized CM-NF.

**Figure 5:**
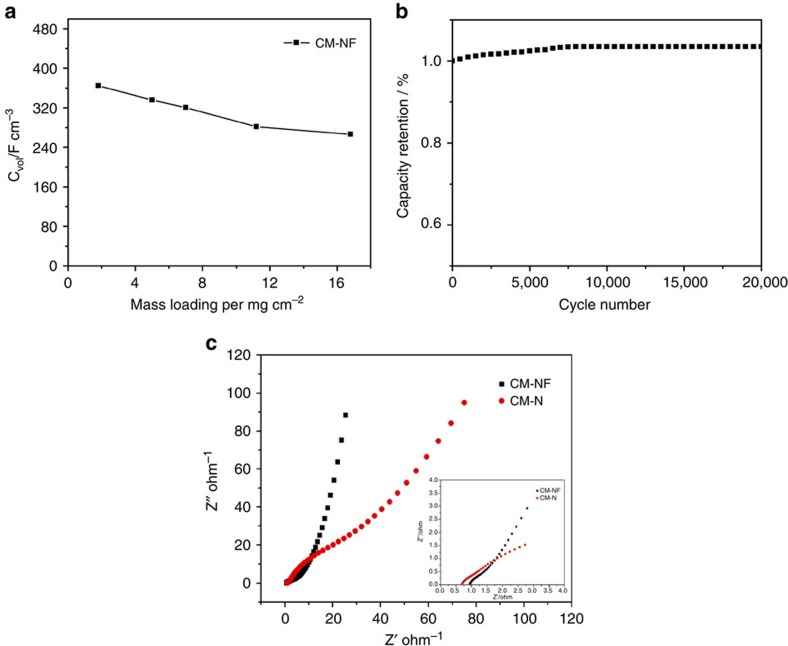
Mass loading and cycling stability. (**a**) Comparison of the volumetric capacitance of the CM-NF electrodes at different mass loading tested in a 6 M KOH solution at a current density of 0.1 A g^−1^ and (**b**) stability evaluation of the CM-NF electrodes in a 6 M KOH solution at a charge current of 5 A g^−1^ for 20,000 cycles; (**c**) Electrochemical impedance spectra (inset: magnified 0–4 Ω region) under the influence of an ac voltage of 5 mV.

The CM-NF electrode also shows exceptional electrochemical performance at very-high mass loadings up to 16 mg cm^−2^, as tested in 6 M KOH. With increased mass loadings, it is expected that the capacitance decreases accordingly because of the increased ion and electron transport resistance/distance^7^,^44^. The volumetric capacitance of CM-NF in 6 M KOH dropped only by 27% (from 365 to 280 F cm^−3^) (See Fig. 5a) as the area-based density increased from 3.2 to 16.8 mg cm^−2^, suggesting immense potential for commercialization for compact energy storage devices. The CM-NF electrode achieves a high volumetric energy density of 18.1 Wh l^−1^ in basic solution. Because of the high volume fraction of the electrodes in the device stack and high mass loading (16.8 mg cm^−2^), the volumetric energy density of the whole supercapacitor could be much higher than that of commercial devices. The volumetric energy density of the supercapacitor could be further improved for an asymmetric hybrid supercapacitor based on CM-NF or in an ionic electrolyte with a higher potential window^12^.

The nitrogen and fluorine co-doping CM electrode also shows excellent rate performance and cyclic stability. The volumetric capacity still maintains an extremely high value above 320 F cm^−3^ at a high current density of 5 A g^−1^ in 1 M H_2_SO_4_ (Fig. 4c). The capacitance retention of the CM-NF retains 64% in KOH and 63% in H_2_SO_4_ as current density increases from 0.1 to 5 A g^−1^, which is significantly higher than that of the CM-N (50% in KOH) and (43% in H_2_SO_4_) (Fig. 4c and Supplementary Fig. 7b). The excellent capacitance retention capability is promising for applications in which a high rate of discharge-recharge is required. We further studied the durability of the CM-NF electrode using galvanostatic charge/discharge measurement to characterize the long-term charge/discharge behaviour at a current density of 5 A g^−1^. As shown in Figs 4d and 5b, the electrode exhibits excellent electrochemical stability without loss of specific capacitance after 20,000 cycles in KOH and 10,000 cycles in H_2_SO_4_, indicating a long-term stability of the electrode. The gradually increased capacitance could be possibly attributed to the improved wettability and active process of the electrode, that is, the continuous diffusion of the electrolyte ions into the ultramicropores/graphitic layers will lead to a gradual increase in the effective charge storage sites of the CM-NF electrode and thus the specific capacitance. This can also be inferred by the *in situ* XRD studies (Supplementary Fig. 8) of the CM-NF electrode tested after 0, 2,500, 5,000 and 10,000 cycles. No apparent phase change was observed and broadening of the (002) peak after 5,000 and 10,000 CV cycles indicated the continuous loss of layer ordering upon ion intercalation.

## Discussion

To demonstrate the superior performance of the nitrogen and fluorine-doped CM electrode, the best results reported for carbon-based electrodes are included into Supplementary Table 2 for comparison with major characteristics of surface area, density, electrolytes, the normalized capacitances and the cycling stability. Clearly, the *C*_vol_ value of the CM-NF is much higher than previously reported carbon-based materials. The ultrahigh volumetric capacitance of the CM-NF electrode is possibly due to synergistic effects of nitrogen and fluorine doping affecting the electron-donor/acceptor characteristics and the charge transfer process upon fluorination and a rapid charge transfer in both acid and base electrolytes (see Fig. 4a and Supplementary Fig. 6b). The pseudocapacitive reactions in both acidic and basic electrolytes can be generally attributed to the electrochemically active functional groups such as pyridine-N, pyridine-N-oxide and pyrrol nitrogen groups. As revealed by previous studies^45^,^46^,^47^, these nitrogen groups play a crucial role in inducing pseudocapacitance by improving the charge mobility of the carbon and by introducing negative charges on the carbon surface, resulting in ion doping/de-doping similar to that observed in conducting polymers. The dominated electrochemically active pyridine and pyrrol-type nitrogens as evidenced by the XPS spectrum (Fig. 3c) and very-high nitrogen doping concentration (∼8–9 at% nitrogen doping for CM-NF and CM-N) result in high capacitance. On the other hand, the existence of ultramicropores may be accessible to small electrolyte ions and favourable for the fast diffusion of electrolyte ions, enabling both Faradaic reactions both at the surface and in the bulk. A sharp increase in capacitance with decreasing pore size has been supported by studies of Gogotsi *et al*., revealing an anomalous increase in volumetric capacitance for microporous carbons with pores <1 nm (ref. 19).

The improved kinetics of ionic transport of both CM-NF and CM-N electrodes in 6 M KOH is clearly evidenced by electrochemical impedance spectroscopy (EIS) with a frequency range from 0.01 Hz to 1 MHz (Fig. 5c and an inset showing EIS at high frequency). The doping of fluorine greatly reduces the charge transfer resistance of electrode, similar to the enhanced electrical conductivity of fluorination observed previously due to the high electronegativity of fluorine atoms. This is consistent with a Density Functional Theory (DFT) calculation (see a DFT calculation in Supplementary Fig. 9 and Supplementary Table 3) showing that fluorine atoms can induce redistribution of charges of N atoms and reduced the gap between Highest Occupied Molecular Orbital (HOMO) and Lowest Unoccupied Molecular Orbital (LUMO) levels for CM-NF, and thus reduce the charge transfer resistance of electrodes (see Supplementary Discussion and Supplementary Methods). The steeper slope of the CM-NF than CM-N in the low-frequency region of the EIS spectra (Fig. 5c) confirms better capacitive behavior as a result of the doping of fluorine. The greater degree of graphitization of the CM-NF may also effectively facilitate the transport of electrolyte ions and high electrical conductivity during the charge/discharge process^48^. The greatly improved charge transfer efficiency is responsible for superior performance of the co-doped electrodes in mitigating capacitance fading at higher current density (over 320 F cm^−3^ for CM-NF versus ∼80 F cm^−3^ for the CM-N).

In summary, we demonstrate an effective strategy of designing high-performance electrodes with ultrahigh volumetric capacitance based on nominally non-porous CMs with co-doping nitrogen and fluorine. The greatly enhanced electrochemical performance, resulting from the pseudocapactive behaviour from heteroatom doping, provides an ultrahigh volumetric capacitance up to 521 F cm^−3^. The incorporation of fluorine is critical, leading to over 100% enhancement in volumetric capacitance as compared with the electrode with nitrogen doping only, and the CM-NF electrode displays exceptional cyclic stability without any capacity loss over 10,000 cycles. The electrode can be synthesized by a scalable, cost-effective approach based on a simple low-temperature solvothermal technique, and the ultrahigh volumetric capacitance and exceptional cyclic stability enable its immense potential for compact energy storage devices with simultaneously high energy and power densities.

## Methods

### Materials synthesis

The CM-NF were prepared using a solvothermal reaction in a stainless steel autoclave (40 ml in total capacity) under autogenous pressure, and a dry glove box with flowing N_2_. In the typical process, 2 g ammonium fluoroborate (NH_4_BF_4_, as the fluorine source) and 1 g hexadecyl trimethyl ammonium bromide (CTAB, as the stabilizer) were mixed into a stainless steel autoclave filled with 4 ml anhydrous CH_3_CN (as the carbon source and nitrogen source) and 24 ml benzene (as the solvent). The autoclave was sealed and maintained at 400 °C for 16 h in a furnace, and then cooled room temperature naturally. The products were collected and washed with distilled water, ethanol and hydrochloric acid several times to remove the impurities. Then the product was dried in vacuum at 80 °C for several hours. CM-N was synthesized under a similar condition without NH_4_BF_4_. The CMs were purchased commercially from Tianjin BTR New Energy Materials Co, Ltd China as control materials for performance comparison with the co-doped carbon spheres.

### Materials characterization

X-ray powder diffraction (XRD) was carried out on a D/max-2500/PC X-ray diffractometer with Cu-*K*α radiation (*λ*=0.15418, nm). Raman spectra of the samples were recorded at room temperature using a WiTec Raman system (Alpha 300) with a × 100 objective lens and a 633 nm He-Ne laser, and also tested using a Princeton Instruments Spectrograph (SP2750) with × 20 objective and 532 nm excitation source. The morphology and elemental information were analysed by scanning electron microscopy and energy-dispersive X-ray spectroscopy (JEOL JSM 7001 F scanning electron microscopy and Oxford-Horiba Inca XMax50 EDX) with the accelerating voltage of 5 kV. The microstructure of the CMs was analysed by a transmission electron microscopy (Hitachi H-7650) operated at 120 kV. High-resolution transmission electron microscopy and selected-area electron diffraction were used to investigate the microstructure using a JEM-2010 transmission electron microscope. XPS measurements were performed on a PHI QUANTERA-II SXM X-ray Photon-electron Spectroscopy. N_2_ sorption analysis was conducted on a TriStar 3020 accelerated surface area equipped with automated surface area at 77 K by using BET calculations for the surface area. In addition, the CO_2_ isotherms were recorded at 273.2 K with a Micromeritics ASAP 2460. SAXS experiments were performed using a NanoSTAR-U (BRUKER AXS INC.) with Cu *K*α radiation (wavelength *λ*=0.154 nm) operated at 40 kV and 650 μA. Two-dimensional SAXS patterns were obtained using a HI-STAR detector. The sample to detector distances were length sample detector (LSD)=1,074 mm. The effective scattering vector **q** (
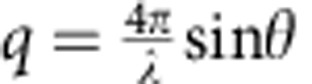
, where 2*θ* is the scattering angle) at this distance ranges from 0.044 to 2.0 nm^−1^.

### Electrochemical measurements

In a three-electrode system, the test electrode was prepared by loading a slurry consisting of 85 wt% active material, 10 wt% carbon black and 5 wt% poly-(vinylidene fluoride) (in *N*-methylpyrrolidone) on a nickel foam and dried at 80 °C for 6 h under vacuum. As-formed electrodes were then pressed at a pressure of 10 MPa and further dried under vacuum at 100 °C for 12 h. Electrodes were obtained by coating an active mass of 2–4 mg onto each current collector (1 cm^2^ area). The sample was used as the test electrode with a platinum foil as the counter electrode. The reference electrode was Hg/HgO in 6.0 M KOH or SCE electrode in 1 M H_2_SO_4_. To prepare the symmetric supercapacitors, a homogeneous slurry containing 85 wt% active material, 10 wt% carbon black and 5 wt% poly-(vinylidene fluoride) (in *N*-methylpyrrolidone) was painted between two pieces of nickel foam with area of 1 cm^2^ and dried at 100 °C in vacuum overnight. The mass loading of the active material was calculated by the mass difference of the nickel foams before and after the loading of active materials. The amount of active materials was 3.7 mg cm^−2^ on the cathode and anode, respectively. There were immersed in the 6 M KOH electrolyte for 3 days at room temperature. CV studies were performed by a CHI 660D electrochemical workstation (Shanghai Chenhua, China) in the potential range of −0.9 to 0.1 V versus Hg/HgO and −0.2 to 0.8 V versus SCE at the scan rate of 10 mV s^−1^. Galvanostatic charge/discharge cycles were measured by a Land cell tester (CT 2001A) at 0.1–5 A g^−1^ over a voltage range of −0.9 to 0.1 V versus Hg/HgO and −0.2 to 0.8 V versus SCE. The specific capacitance *(C*_s_) of the systems was calculated according to the following equation:


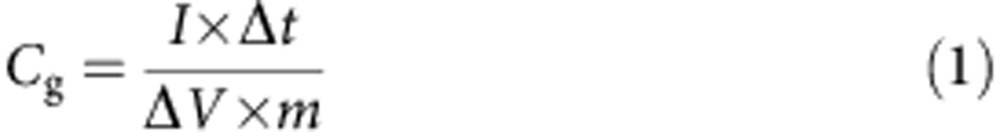



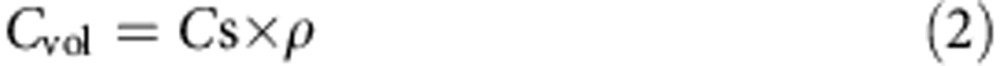



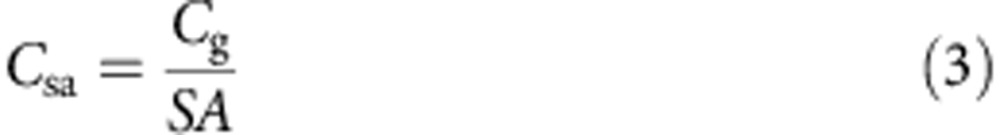



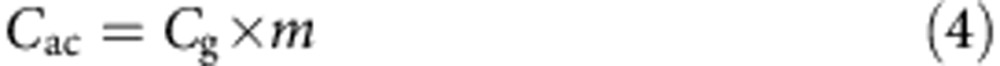


where *C*_s_ (F g^−1^) is the gravimetric-specific capacitance, *C*_vol_ is the volumetric-specific capacitance, *C*_sa_ is the specific capacitance per *SA*, *C*_ac_ is the area-based specific capacitance, *I (A)* is the charge/discharge current, *ΔV* (V) stands for the potential window within the discharge time Δ*t* (s), and *m* (g) corresponds to the amount of active material on the electrode, *ρ* is the density of our samples and *SA* is the surface area of our samples. All the experiments were conducted at room temperature (25±1 °C).

## Additional information

**How to cite this article:** Zhou, J. *et al*. Ultrahigh volumetric capacitance and cyclic stability of fluorine and nitrogen co-doped CMs. *Nat. Commun*. 6:8503 doi: 10.1038/ncomms9503 (2015).

## Supplementary Material

Supplementary Information Supplementary Figures 1-9, Supplementary Tables 1-3, Supplementary Discussion, Supplementary Methods and Supplementary References
