# Supplementary material for: Ultrahigh volumetric capacitance and cyclic stability of fluorine and nitrogen co-doped carbon microspheres
Source: Nat Commun. 2015 Sep 29;6:8503. doi: 10.1038/ncomms9503 (PMC4598840; doi:10.1038/ncomms9503)
Supplement: Supplementary Information — Supplementary Figures 1-9, Supplementary Tables 1-3, Supplementary Discussion, Supplementary Methods and Supplementary References [file ncomms9503-s1.pdf]

Supplementary Figures

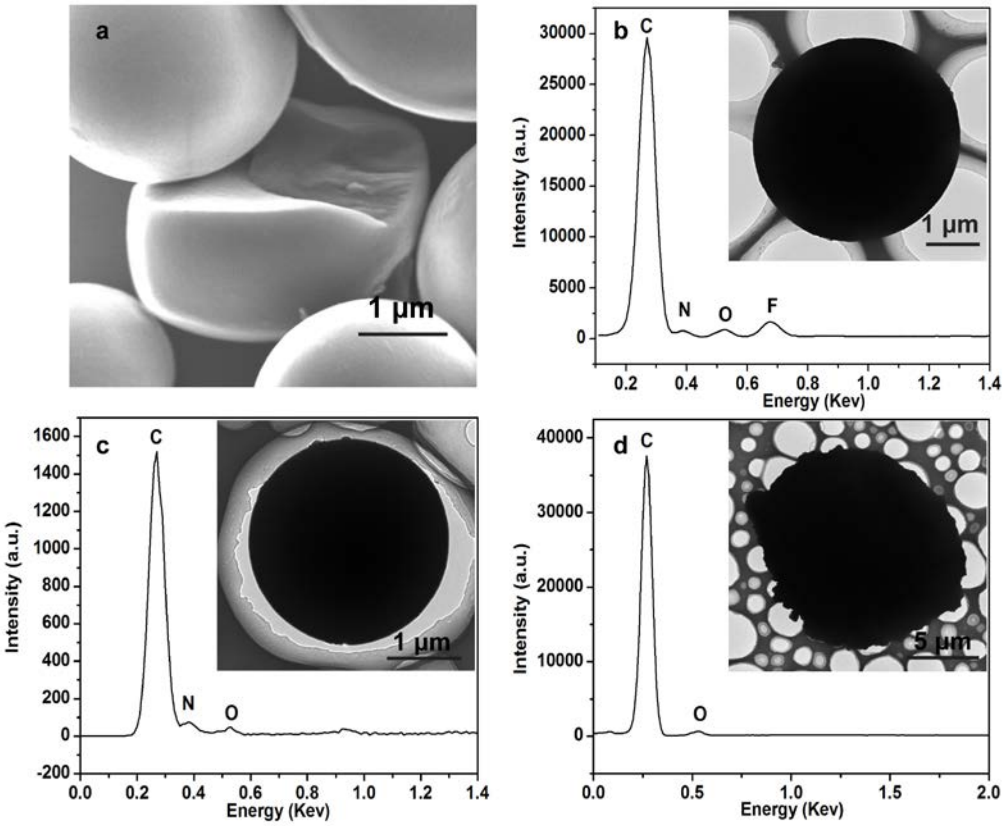

Supplementary Figure 1: Microstructure, morphology and chemical composition of the carbon microspheres: (a) A SEM image of the CM-NFs; and EDS spectra of CM-NFs (b), CM-Ns (d) and CMs (e). The insets in Figs. S1b-d are the corresponding TEM images.

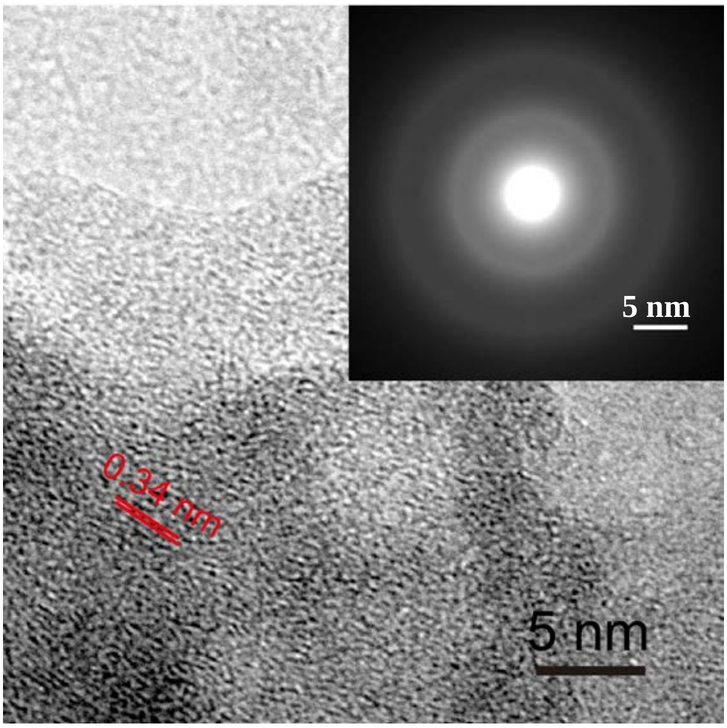

Supplementary Figure 2: HRTEM image of the CM-NFs. Inset: Selected-area electron diffraction pattern of the CM-NFs.

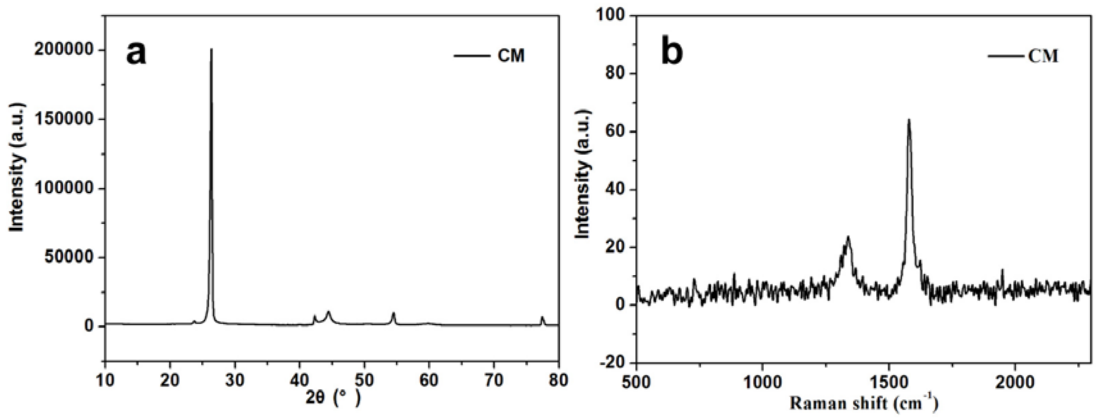

Supplementary Figure 3: (a) XRD patterns and (b) Raman spectra of the commercial pure CMs

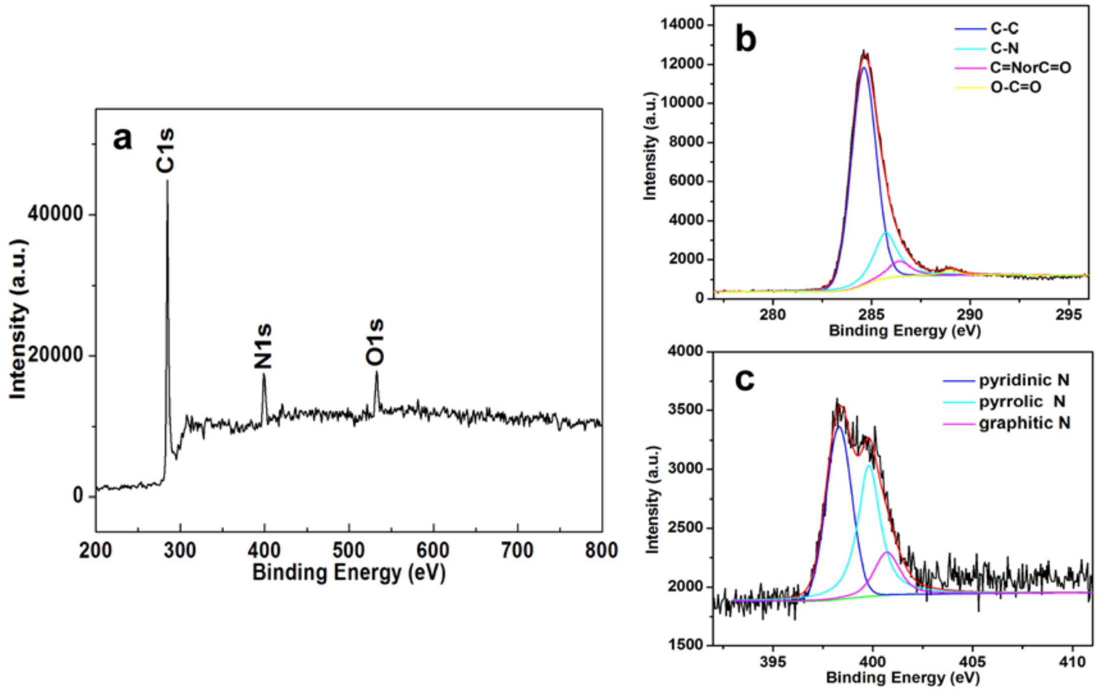

Supplementary Figure 4: (a) XPS survey spectrum of CM-Ns; and (b-c) High-resolution XPS spectra of C 1s, and N 1s of CM-Ns, respectively.

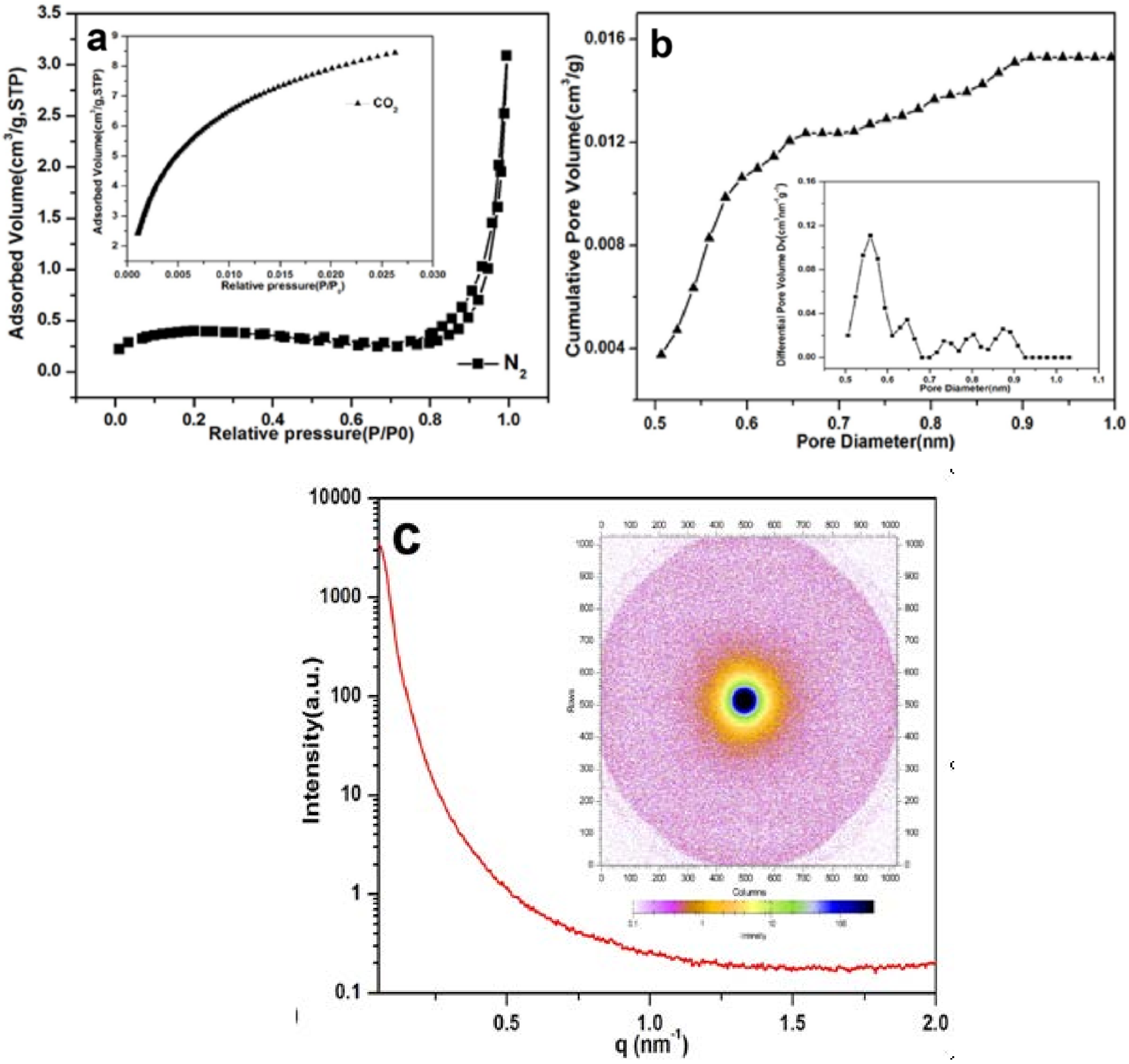

Supplementary Figure 5: (a) High-resolution, low-pressure N_2 (77.4 K) isotherms. (Inset) The CO_2 (273.2 K) isotherm. (b) Cumulative pore volume and (inset) pore-size distribution for CO_2 (calculated by using a slit pore NLDFT model). (c) Small-angle x-ray scattering (SAXS) patterns of synthesized CM-NFs.

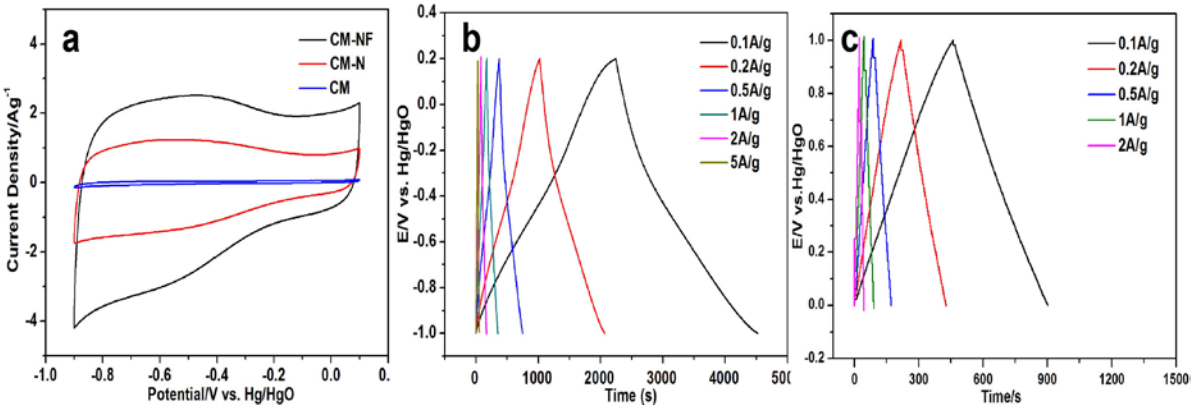

Supplementary Figure 6: (a) CV curves of CM, CM-N, and CM-NF samples in a 6 M KOH solution at a scan rate of 10 mV s^{-1}; (b) Galvanostatic charge/discharge curves of CM-NF samples in a 6 M KOH solution with different current densities using three electrodes; (c) Galvanostatic charge/discharge curves of CM-NF samples in a 6 M KOH solution with different current densities using two electrodes.

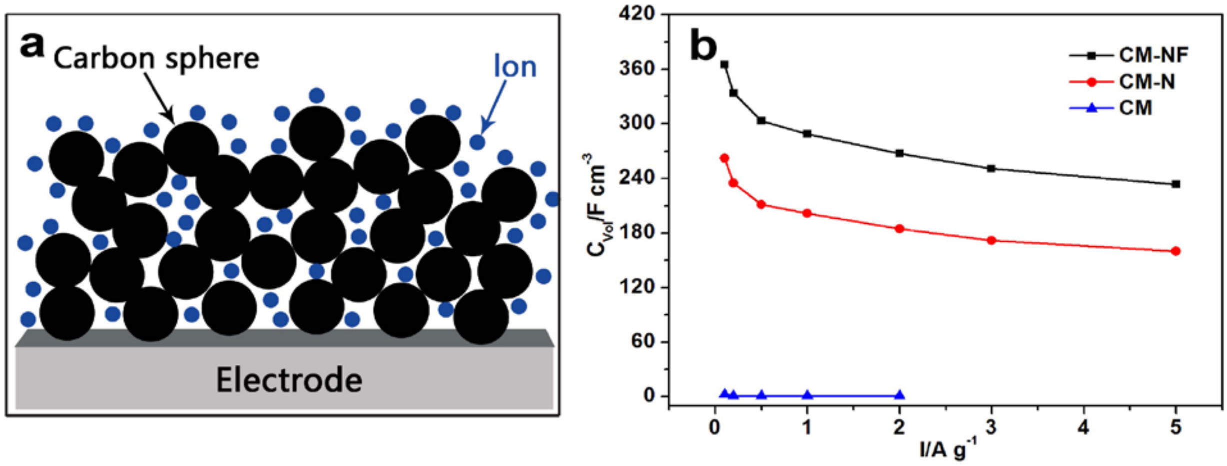

Supplementary Figure 7: (a) Schematic model comparing the ion diffusion for CM-NFs; (b) corresponding capacity retention at the current density from 0.1 to 5 A g^{-1};

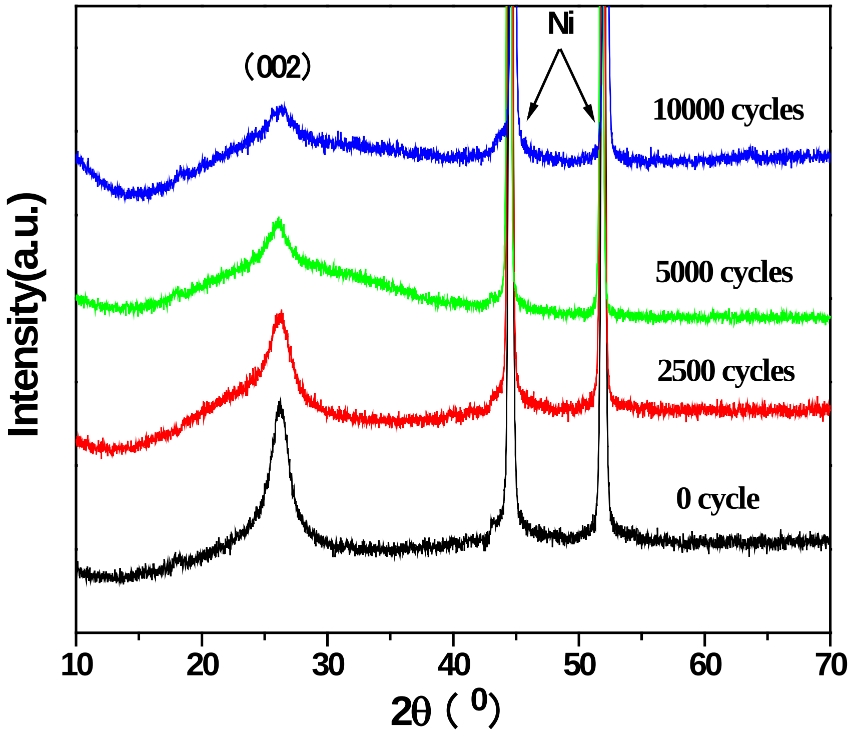

Supplementary Figure 8: XRD patterns of the CM-NF electrodes on Ni foam after cycling test for 0, 2500, 5000 and 10000 cycles at a charge current of 5A g^{-1} in 6M KOH. No phase change was observed. Broadening of the (002) peak suggested further reduction of the layer ordering of the carbon spheres upon ion intercalations with cycling.

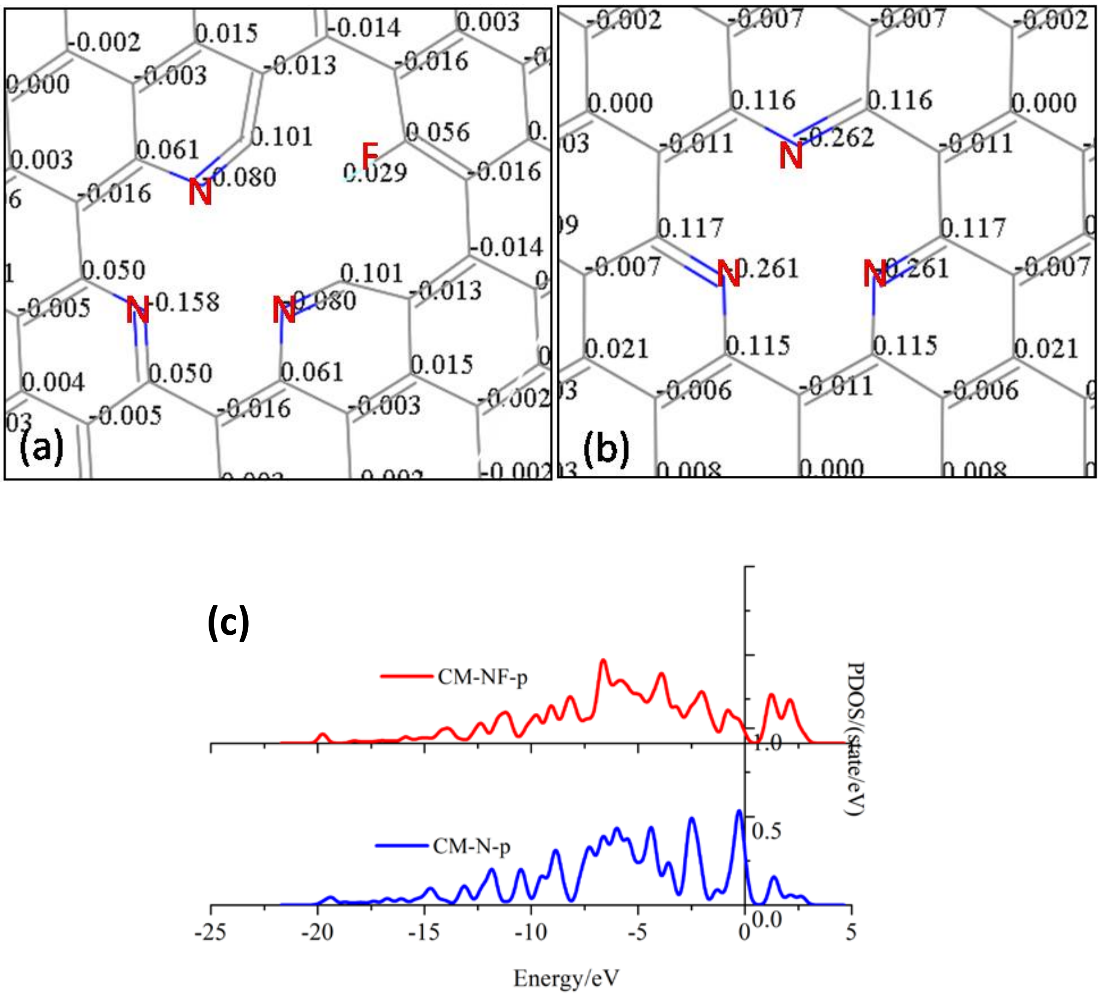

Supplementary Figure 9: DFT calculation of the charge distribution of CM-NFs (a) and CM-Ns (b) and DOS (c).

Supplementary Tables

Supplementary Table 1: Composition of CM-NFs, CM-Ns, CMs and their surface areas ([a]Derived from XPS. [b]Derived from EDS elemental analysis. [c]BET surface)

Sample	C^a	N^a	O^a	F^a	C^b	N^b	O^b	F^b	$S_{BET}{}^c/m^2\,g^{-1}$
**CM-NF**	**83.6**	**8.76**	**4.24**	**3.4**	**85.6**	**7.83**	**3.05**	**3.52**	**1.4**
**CM-N**	**84.36**	**9.89**	**5.75**	**0**	**86.2**	**9.17**	**4.63**	**0**	**1.9**
**CM**	**95.3**	**0**	**4.7**	**0**	**96.3**	**0**	**3.7**	**0**	**1.0**

Supplementary Table 2: Performance of selected porous carbon materials for ECs

Item	density /g cm^{-3}	BET/ m^2 g^{-1}	electrolyte/ mol L^{-1}	C_s/F g^{-1} (I/A g^{-1})	C_{vol}/ F cm^{-3}	Cycling Stability	Ref
Laser scribed graphene	0.048	1520	H_2SO_4 (1.0)	202 （1.0）	9.7	with 3% deterioration after 10000 cycles	1
Graphene hydrogel	0.05	1050	H_2SO_4 (1.0)	258 (0.3)	12.9	without degradation after 2000 cycles	2
Oriented graphene hydrogel	0.069	No data	H_2SO_4 (1.0)	215 （0.1）	14.8	with 3% deterioration after 10000 cycles	3
Chemically modified graphene	0.5	705	KOH (5.5)	135 （1.33）	67.5	No data	4
Commercial activated carbon	0.5~0.7	1500~ 2000	KOH (6.0)	160~ 200	80~ 110	No data	5
EM-CCG film	1.33	166.8	H_2SO_4 (1.0)	191.7 (0.1)	255.5	with 5% deterioration after 300 Hours	6
CM-N	1.71	1.9	KOH (6.0)	153.3 （0.1）	262	without degradation after 10000 cycles	This work
CM-NF	1.93	1.4	KOH (6.0)	189 （0.1）	365	without degradation after 20000 cycles	This work
CM-NF	1.93	1.4	H_2SO_4 (1.0)	270 （0.2）	521	without degradation after 10000 cycles	This work

Supplementary Table 3: Geometry parameters and HOMO and LUMO gaps (Eg) for the CM-NF and CM-N systems

Parameters	CM-NF	CM-N
HOMO/eV	-3.93	-4.718
LUMO/eV	-3.737	-3.404
Eg/eV	-0.1925	-1.314
$d_{C\text{-}N}$/Å	1.311	1.344
$d_{C\text{-}C}$/Å	1.407	1.456

Supplementary Discussion

To understand reversible Faradaic redox reaction, density functional theory (DFT) calculations were performed. We constructed a single carbon layer to understand the edge N and F doping and electrochemical properties of the materials. The reconstructed defect in vicinity doping atoms and redistribution of charges of F atom and N atoms are shown in Supplementary Figure 9. From Supplementary Figure 9a, it can be seen that F doping induces redistribution of charges of N atoms in CM-NF. N atoms takes on a mixed valence state between -0.158 and +0.101, which is very favorable to promote the variance of DOS around N atoms with charge-discharge.

The PDOS of 2p orbitals of CM-NF and CM-N are shown in Supplementary Figure 9c. The number of states in unoccupied band may correlate to the capacitance of materials. From Supplementary Figure 9c, it can be seen that the states in unoccupied band of CM-NF is larger than that of CM-N, indicating a better electronic acceptance of CM-NF. The calculated energy gaps between HOMO and LUMO are listed Supplementary Table 3. CM-NF has a smaller energy gap than CM-N. The energy gap and the conductivity can be connected by effective mass. Thus, CM-NF could possess higher conductivity due to its small energy gap, which is beneficial to improve the electronic transition and its capacitance. The doping of fluorine greatly reduces the charge transfer resistance of electrode and enhances the degree of graphitization, all these are responsible for the superior capacitive performance of the N and F co-doped CM.

Supplementary Methods

We performed DFT calculation using a periodically repeated slab model with the DMol3 Package to investigate a nitrogen and fluorine doped carbon microsphere. We used graphene, i.e., graphite(0001), instead of carbon microsphere in the DFT calculations. The carbon microsphere surface can be approximated as graphene on a nanoscale. Carbon microsphere consists of stacking graphene layers via interlayer van der Waals interactions. A structure model for monovacancy, defective graphene was composed of 127 atoms in a 19.68×19.68×20 Å orthorhombic supercell. Full geometry optimizations were performed for all the models, and all atoms were free to relax. All calculations were performed using the DFT semicore pseudopots approximation to replace the core electrons with a single effective potential. The double numerical plus polarization (DNP) basis sets were used for the valence orbitals and provided accuracy, and DNP basis sets are comparable to 6-31G** sets. The exchange correlation contributions were described within the Perdew-Burke–Ernzerhof (PBE) exchange-correlation functional.

Supplementary References

1. El-Kady, M. F., Strong, V., Dubin, S. & Kaner, R. B. Laser scribing of high-performance and flexible graphene-based electrochemical capacitors. *Science* 335, 1326–1330 (2012).
2. Wu, Q., Sun, Y. Q., Bai, H. & Shi, G. Q. High-performance supercapacitor electrodes based on graphene hydrogels modified with 2-aminoanthraquinone moieties. *Phys. Chem. Chem. Phys.* 13, 11193–11198 (2011).
3. Yang, X. W., Zhu, J. W., Qiu, L. & Li, D. Bioinspired effective prevention of restacking in multilayered graphene films: towards the next generation of high‐performance supercapacitors. *Adv. Mater*. 23, 2833–2838 (2011)
4. Stoller, M. D., Park, S. J., Zhu, Y. W., An, J. H. & Ruoff, R. S. Graphene-based ultracapacitors. *Nano Lett.* 8, 3498–3502 (2008).
5. Simon, P. & Gogotsi, Y. Capacitive energy storage in nanostructured carbon electrolyte systems. *Acc. Chem. Res*. 46, 1094-1103 (2013)..
6. Yang, X. W., Cheng, C., Wang, Y. F., Qiu, L. & Li, D. Liquid-mediated dense integration of graphene materials for compact capacitive energy storage. *Science* 341, 534–537 (2013).
